# A systematic literature review on how consumer and community involvement have shaped and influenced pre-pregnancy care interventions for women with diabetes

**DOI:** 10.1186/s12884-024-06951-9

**Published:** 2024-11-13

**Authors:** Rachel Hicks, Tinashe Dune, Veronica Gu, David Simmons, Freya MacMillan

**Affiliations:** 1 Macarthur Clinical School, Western Sydney University, Sydney, Australia; 2 Translational Health Research Institute, School of Health Sciences, Western Sydney University, Sydney, Australia; 3 School of Medicine, Western Sydney University, Locked Bag 1797, Campbelltown, NSW 2751 Australia

**Keywords:** Pre-existing diabetes, Type 1 diabetes, Type 2 diabetes, Pre-pregnancy Care (PPC), Consumer Community involvement (CCI), Communication, Interventions

## Abstract

**Background:**

Diabetes and pregnancy studies have found better outcomes when interventions were developed with consumer (individuals with lived experience of diabetes) and community involvement. When consumers are central to development and delivery of interventions, study participants have better engagement and outcomes, particularly for individuals from culturally and linguistically diverse (CALD) and/or lower socio-economic backgrounds. Our study aims to examine the scope of consumer and community involvement (CCI) in the construction and implementation of pre-pregnancy care (PPC) interventions and discuss a framework for consumer-lead intervention development.

**Methods:**

A systematic literature review was conducted, examining 3 electronic databases. A meta synthesis analysis of tabulated data summarized in a literature matrix was undertaken with a phenomenological approach to develop a Pre-Pregnancy Care CCI-Driven Intervention Framework.

**Results:**

Overall, 4642 papers were identified, with 29 meeting inclusion criteria. The meta-synthesis and literature matrix identified several common themes across previous studies. These were: barriers to accessing (PPC) such as negativity and stigma in care from behaviours, attitudes and perceptions of HCPs; limited appointment availability not aligning with work and family commitments; fear of losing a “normal” pregnancy journey; awareness of risk but unwillingness to discuss if consumers have not established trust with HCPs; inaccessibility to CALD appropriate PPC and contraception; and digitisation of PPC information resources including peer support and social media. From these results, a PPC Consumer-Driven Intervention Framework for Women with Pregestational Diabetes was developed with recommendations.

**Conclusion:**

Consumers have been under-involved in the majority of previous developments and implementation of interventions for women with diabetes and pregnancy, and their representation as stakeholders in interventions is paramount to the longevity of intervention outcomes. To assist community involvement in diabetes pregnancy intervention design and delivery we created a new framework, for improving clinical and social outcomes in healthcare, empowering relationships between HCPs and consumers, and highlighting the value of lived experience and women-centred care for increased community engagement.

## Background

Research on diabetes and pregnancy interventions to reduce adverse pregnancy outcomes including congenital malformations among women with pre-existing diabetes has included focusing on the implementation of high dose folic acid intake, near-normalised glycaemia and establishment of pre-pregnancy counselling and specialist diabetes services [1]. The majority of studies determining the impact of medical interventions are developed based on clinically-measured outcomes and reporting requirements and measurements within healthcare frameworks. However, there is increasingly a move within healthcare research generally, and specifically within diabetes research, to include active consumer and community input and feedback in the development and implementation of interventions due to the associated increased longevity of outcomes [2].

A previous systematic review examining the effectiveness of pre-pregnancy care (PPC) (healthcare services for women with diabetes of child-bearing age for improving maternal and perinatal outcomes) found that interventions with consumer and community involvement (CCI) with PPC resulted in significant and clinically beneficial effects. Notably, congenital malformation risks reduced by 71%, lower HbA1c in the first trimester (average − 1.27%), a 15% reduction in pre-term delivery, as well as a reduction in risk of preterm delivery by 54% [3]. However, this review reported low uptake of PPC, especially among consumers of low socioeconomic status, multiple barriers to consumers engaging with PPC due to lack of knowledge, as well as pre-conceived attitudes and behaviours towards messages from healthcare professionals about diabetes, pregnancy and risk.

Consumer (individuals with lived experience) and community involvement in interventions is important for reaching intended target audiences, regardless of its impact on efficacy. For example, ethnographic studies of the lived experiences of women with diabetes on their pregnancy and postpartum journeys have shown that when the consumer is central to the successful development and delivery of an intervention, the target consumer is more likely to have long-term engagement and increased successful outcomes [4]. However, there were limitations to this study including being a single-site study in one country (Sweden), with limited participants (five at recruitment, and three who participated in the entire intervention). This previous study also focused on the within pregnancy journey and not pre-conception perceptions. Whilst consumers indicated a web-browser-based support intervention was difficult to navigate and had minimal connectivity to clinical services, the study itself served as a vehicle to demonstrate consumers as equals in intervention development [4]. As such, the proposed reach and engagement of studies in the real-world is greater when consumers are involved in the co-development of projects and resources for their benefit [2]. Therefore, intersectionality of CCI (for social validity) and healthcare service input (for clinical validity) is important in developing interventions that are more likely to be engaging, relevant and therefore ultimately successful, especially in the context of culturally and linguistically diverse (CALD) and/or low socio-economic audiences. CALD and low socio-economic communities in particular have demonstrated varied levels of engagement depending on intervention study type.With the COVID-19 pandemic, and move to telehealth services [5], an increase in engagement with digital support methods has supported easier access to translation and peer support in consumers’ native languages [6]. However, many interventions and associated services also include a face-to-face component based on cultural importance of community representation and consumer preference [7–9]. Therefore, examining consumer perspectives and preferences in interventions and their development is a potential untapped research area in PPC research for women with diabetes.

## Methods

### Objective

To examine the scope of consumer and community involvement (CCI) in the development and implementation of previous PPC interventions for women with pre-existing diabetes, and develop a set of recommendations and framework for delivering optimal interventions for improved/increased pregnancy planning or effective contraception use among women with type 1 and type 2 diabetes.

### Research questions

From the consumer perspective, what components of PPC interventions are required for successful reach (including diverse communities), ongoing engagement and long-term community commitment? What would a CCI framework for the development of PPC interventions look like based on important components identified from previous PPC interventions?

A systematic review was undertaken following PRISMA 2020 guideline for systematic reviews [10]. Searches were made in OVID-Medline, CINAHL and ProQuest Central databases (Communication Abstracts, Education Research Complete, Health Business Elite, Health Source - Consumer Edition, Health Source: Nursing/Academic Edition, APA PsycInfo, SocINDEX with Full Text). These databases were chosen to ensure a thorough search of current literature across clinical, social, psychological and communication databases was conducted.

A scoping search was undertaken using a population, concept, and context framework [11] to develop a search strategy including relevant keywords and their synonyms relating to the population of interest (women with pre-existing diabetes either in the preconception/pre-pregnancy planning stage or early pregnancy), the concept of consumer input and for the context of planning pregnancy or contraception. Appendix 1 includes the full search strategy used for OVID-Medline. Search terms were adjusted where necessary in the other databases (e.g. if different indexing was used). Database results were downloaded and screened at title/abstract followed by the full text level in Endnote (version x9) against inclusion criteria.

Inclusion criteria were: full-text journal articles in English including qualitative or mixed methods approaches with a qualitative component that were either an intervention protocol, intervention outcomes or consumer input analysis paper. Studies had to focus on pregestational diabetes PPC interventions that reported engaging with consumers or community in any phase of its development. Exclusion criteria of papers were: studies that focused primarily on experiences and feedback from clinical staff rather than consumers and community; studies focusing on women during pregnancy, unless they demonstrated a clear PPC component; studies focusing on women with Gestational Diabetes Mellitus due to the differentiation in diagnosis/criteria and longevity of diagnosis in comparison to Type 1 and Type 2 diabetes. Study inclusion and exclusion criteria are detailed in Table 1.

**Table 1:** Study Selection (conducted September 2021)

Inclusion criteria	• PPC Interventions in women of reproductive age with pre-existing diabetes (type 1 or 2), either not pregnant or in the early stages of pregnancy when accessing the services were required to be examined in the research presented. • Interventions included education, clinical services, counselling (relating to folic acid intake, insulin and blood glucose monitoring, contraception, and peer support/online/app referrals). • Articles referencing the consumer role in PPC studies and reporting of qualitative and quantitative data from consumers (women with pre-existing diabetes either in the preconception/pre-pregnancy planning stage or early pregnancy) • Language associated with communication or consumer interventions (i.e., Care, support, education, perceptions) • Studies needed to report on pre-pregnancy/prenatal planning and care services, or communication practices between patients with diabetes and healthcare professionals in association with pregnancy or pregnancy planning in the context of their diabetes care.
Exclusion criteria	• Papers with no examination of the consumers/were purely clinical in nature, papers which focused on postpartum care, and interventions recruiting only pregnant/postpartum women or majority (90% or more pregnant women) or children (< 18-year-old). • Postpartum/postnatal-only studies. Whilst it is acknowledged how vital research is on postpartum interventions and improving outcomes for women with diabetes, this research project focuses on the early pregnancy journey interventions. • Studies recruiting women with Gestational Diabetes/GDM. due to the differentiation in diagnosis/criteria and longevity of diagnosis of GDM in comparison to Type 1 and Type 2 diabetes, studies of GDM were excluded

The first author screened all database returns (RH) and extracted data, manually. A quality assessment of studies was then completed using the Critical Appraisal Skills Program (CASP) Qualitative Studies Checklist (Appendix 2) which was independently evaluated (VG) and reviewed by the authors [12] to assist with interpretation of findings in terms of the quality of studies (including examination of mixed-methods study qualitative components). A literature matrix (Table 2) with study type, methodology, location of study, consumers involved, summary of study results and major themes (including consumer feedback), and extent of CCI was then used to summarise extracted data. Any papers which were unclear of meeting inclusion or exclusion criteria, were discussed with all authors for final decision.

**Table 2:** Systematic review literature matrix (completed April 2022):

Author	Year	Study Type	Context (Study Location)		No. Participants	Method		Results and Major Themes	Consumer/Community involvement	
Murphy et al.	2009	Intervention study with qualitative evaluation	UK		29	Qualitative open-ended survey and thematic analysis		-Women who do not engage with PPC generally had an understanding of the benefits of PPC/risks associated with sub-optical glycaemic control and unplanned pregnancy. - Almost half of the women deliberately discontinued contraception and conceived quicker than anticipated. - Attendance was hindered based on fear of a negative experience due to “unplanned pregnancy”, anticipated negative response from HCPs, a desire for a “normal pregnancy” with less medical intervention, and the logistics of attending due to multidisciplinary care. -More integrated diabetes/reproductive health and contraception advice is needed - further investigation on how communication between HCPS and women with diabetes can be improved. - HCPs need to work with women and their partners during routine care to try and identify barriers/strengthen communication between them and HCPs; encouraging PPC attendance based on trust and recommendation of their treating team.	Very involved - thematic analysis of women with diabetes, feedback on why they didn’t attend PPC/how it can be improved	
Lavender et al.	2010	Intervention study with qualitative evaluation	UK	22			Hermeneutic phenomenological - focus groups and interviews with interpretive analytical approach	- Main barrier to attending PPC is time commitments/disruption to work/family and potentially disclosing what should be a private affair to their workplace to get consideration to attend the large number of appointments. - Lack of sensitivity from HCPs about understanding the disruption and sacrifices that consumers make to be able to attend. As such, a social model of care and flexibility in PPC delivery is suggested. - Consumers struggled with the feeling of relinquishing personal control with diabetes and pregnancy experience, and that they had haphazard PPC. Frustration with losing control to a system of care that caused huge disruption to their lives via appointment attendance requirements. However, they accepted surrendering of their control to HCPs and others who held their trust. All expressed disappointment that at appointments, the focus was overridden with diabetes management rather then pregnancy/pregnancy planning. - Only those planning pregnancy/multigravida with pregnancy or pregnancy loss stated they understood importance of PPC. Apathy to PPC based on lack of information/emphasis from HCPs on importance of attendance. - Women should be supported to optimise their experience as well as clinical outcomes. Convergence of professional roles needs consideration; individual members of multidisciplinary diabetes teams should provide a unique and complementary contribution that needs to be accessible and responsive to women (including CALD cohorts)		Involved - consumers were part of focus groups and interviews which were then analysed to determine study findings.
Rodgers Fischl et al.	2010	Intervention study with qualitative evaluation	USA	88			Intervention participation study - experimental randomised-controlled design. Self-administered questionnaires with comparative analysis and mixed-modelling methods.	- Adolescent women with diabetes were placed in the READY-Girls program intervention, viewed a CD-ROM/Book and met with a nurse, of which their knowledge/attitudes/behaviours regarding diabetes and sexuality/PPC was assessed. - The results showed benefit and sustainability at a low-cost, with at least 9 months of successful outcomes and evidence-based more empowered young women with diabetes. - Consumers who engaged with program did request additional PPC resources and information from HCPs involved, as they had established relationship of trust.		Involved - consumers participated in the intervention, however were not part of creating the intervention or able to provide feedback.
Schwarz et al.	2010	Intervention study with qualitative evaluation	USA	89			Questionnaires and descriptive statistical analysis	- Study aimed to assess beliefs, perceived access and practices of contraception in adolescent women with diabetes. Results showed half of the participants were engaging in sexual activity without contraception at a time they were trying to avoid pregnancy, and less than half had discussed birth control options with HCPs. - Over a third felt birth control options were very limited for women with diabetes, and over half stated they felt comfortable asking HCP for birth control. Consumers reported that they worry a lot about becoming pregnant, however most had not been told about or used LARC and were not comfortable asking HCPs for birth control options or disclosing sexual activity. Social desirability bias can result in consumers under-reporting sexual activity to HCPs, as it may be seen as inappropriate to their culture/religion/age.		Involved - consumers were part of focus groups and interviews which were then analysed to determine study findings.
Spence et al.	2010	Intervention study with qualitative evaluation	UK	24			Focus groups, followed by content analysis of transcriptions	- Awareness of rationale of PPC only seen in women who were pregnant or who actively sought PPC. Uncertainty of what PPC was/timeframe to access it, and many cited barriers to accessing PPC due to unsupportive HCPs, busy clinics and perceived stigma/social stereotypes from HCPS. - Five main themes: knowledge; quality of relationships with healthcare professionals; organisation of care; the impact of beliefs/attitudes on advice giving; and women’s attitudes to pre-pregnancy care advice. Study shows that guidance for PPC needs to be given to consumers in a motivating, supportive manner and communicated early on, rather than at a perceived “right age/marital status” - as most may be sexually active prior to this. - HCPs were seen as authoritative and unsupportive to consumer needs/ limited continuity of care made it difficult to establish relationships with HCPs and access PPC.		Involved - consumers were part of focus groups and interviews which were then analysed to determine study findings.
Lawrence et al.	2011	Systematic Review	USA	0 (however paper examined interventions with consumer involvement)			Systematic Literature Review	- Women with pre-existing diabetes need access to reliable woman-friendly information about PPC and management of diabetes during pregnancy. Women recalled hearing messages about pregnancy when they were young girls, including that they may never be able to have children and that they would need to go to a major city to receive their care. - Perceptions/expectations of women with diabetes during pregnancy may effect psychological response to pregnancy and beyond. PPC should be added into ongoing health-care encounters women of child bearing age and adolescent women with diabetes, regardless of their intention to become pregnant at the time of any specific health-care encounter. - Women’s use of PPC is influenced by their previous health-care experiences, in addition to their access to care and knowledge of the importance of preconception care. HCPs need to act in positive health behavioural promotion to give consumers confidence in diabetes and pregnancy self-management and efficacy - that she is doing what is best for herself and her child.		Not involved directly with study, however study examined literature where consumers provided previous experiences.
Shawe et al.	2011	Intervention study with qualitative evaluation	UK	107 for Survey, 16 for interviews			Mixed methods - questionnaire syrvey, semi-structured qualitative interviews	- Study examined factors that promoted or discouraged contraception to women with diabetes, which showed they are likely to receive minimal advice about contraceptive options. - Over a third had not received any contraceptive advice from any HCP, with over half getting contraceptive advice from their GP. Many, due to cultural/family pressure followed natural family planning/calendar as they did not want hormonal intervention with contraception. - Many did not understand the safety/risk of hormonal contraception, and study showed a worrying communication gap between consumers and HCPs in relation to contraception and making an informed decision on options.		Involved - consumers were part of focus groups and interviews which were then analysed to determine study findings.
Sparud-Lundin et al.	2011	Intervention study with qualitative evaluation	Sweden	105			Explorative descriptive content analysis via survey	-Study explored internet usage, needs and expectations among women with diabetes of childbearing age. Majority indicated they are active participants on social websites, especially in relation to accessing information and support on childbearing and diabetes. - Many consumers seek diabetes-related information and support via the internet, particularly before and during pregnancy. Topics such as diabetes-related issues, diabetes devices, dietary advice and social support were the main areas women sought information on in this setting. - Consumers express a need for studies with CCI to have components of online support groups, whether in the capacity of peer support or online support from HCPs The study highlights areas of development including facilitating access to reliable information, interactive support with other consumers and HCPs and social networking/peer support options.		Quite involved - consumers demonstrated their internet usage to provide context for future intervention development related to technology.
Holmes et al.	2012	Intervention study with qualitative evaluation	UK	97			Questionnaire and focus groups	- Women reported logistical barriers to accessing PPC care and information - consideration of embarrassment from adolescents (including with parental attendance), preconceived social stereotypes from HCPs, and busy clinics/lack of continuity of staff should be noted when preparing future interventions. - A DVD was created to determine if it would increase women’s’ knowledge towards PPC and change attitudes. Results show the DVD improved self-efficacy for contraception usage and accessing PPC. Knowledge of planning pregnancy and risks increased significantly after viewing of the DVD. - All women with diabetes, regardless of age/marital status/type of diabetes, should receive guidance about planning pregnancy in a motivational, positive and supportive manner - the language and presentation of risk needs to be carefully constructed to establish and maintain trust.		Involved - women participated in the intervention where their attitudes/knowledge was assessed. However, they were not part of intervention creation.
McCorry et al.	2012	Intervention study with qualitative evaluation	Northern Ireland	14			Semi structured interviews and qualitative analysis (phenomenological approach)	-The perspectives of women interviewed towards their diabetes PPC focused on four themes: (1) the emotional complexity of childbearing decisions, (2) preferences for information related to pregnancy, (3) the importance of being known by your HCP and (4) frustrations with the medical model of care. -Women expressed that having a comfortable, trusting relationship with HCPs who recognised the complex nature of their diabetes and acknowledged the women’s experience was far more valuable than the HCP’s setting or medical background. Relational continuinity was highly valued and when present, resulted in better health outcomes and adherence to goals set in collaboration with the women and their HCPs - Many respondents stated their anxiety in relation to being in control of glucose prevented them from seeking early pregnancy care. - It is imperative for a supportive, trusting relationship between HCPs and women with diabetes and continuity of care in order to provide care that meets the needs of the patient.		Very involved - study focused on interviews with consumers on PPC perspectives.
Sapiano et al.	2012	Intervention study with qualitative evaluation	Malta	37			Self-administered questionnaire, followed by intervention and follow-up questionnaire	-Study aimed to asses knowledge/awareness of PPC of Maltese women of reproductive age with T1D. Results showed a significant lack of awareness of PPC, with 70% not having any knowledge of PPC before the intervention. As such, there is a lack of awareness of the importance of PPC to reduce adverse outcomes in pregnancy and diabetes. - Consumers understood some components of PPC requirements (smoking cessation, glucose management and retinopathy management), and most preferred to learn about PPC from their doctor/specialist, followed by via internet. - Negative feelings about HCPs were expressed by 35% of women with unplanned pregnancies and by 6% of women with planned pregnancies.		Minimal involvement - responses from online self-report survey with no follow-up or space for feedback.
Komiti et al.	2013	Intervention study with qualitative evaluation	Australia	123			Questionnaire	- Aim of study was to further understanding of factors that contribute to PPC update, particularly attitudes and beliefs towards PPC from consumers that were examined using models of health behaviour. - Only perceived benefits were associated with PPC uptake, and having exposure to a greater number of messages/cues related to PPC (especially internet) was a significant factor of, as well as older age or not having children. - More rigorous intervention efforts were recommended for younger women, women with children, CALD women, from rural regions, and with multiple conditions including mental health		Minimal involvement - responses from questionnaire to determine PPC behavioural attitudes, with no follow-up
Spence et al.	2013	Intervention study with qualitative evaluation	UK	97			Questionnaire and focus groups	-A DVD was created by a professional advisory group (HCPs) and reviewed consumers to determine if it would increase women’s’ knowledge towards PPC and change attitudes. -The video includes interviews from women with lived experience of diabetes/pregnancy with evidence-based commentary on the benefits of planned pregnancy -Consumers evaluated the resource and rated the content high in terms of quality, content and interest, therefore was deemed a highly acceptable PPC resource.		Involved - consumers were part of evaluation focus groups and interviews which were then analysed to determine study findings.
Egan et al.	2015	Systematic review and intervention study with qualitative evaluation	Ireland	0 (but paper is CCI protocol: recommend 180/30 service users)			SLR, outcomes prioritised via Delphi survey, meeting to finalise outcomes.	Study examined PPC and outcomes in previous studies via SR, with stakeholder meeting (including consumers) to finalise outcomes.		Not involved directly with study, however findings from previous PPC presented to consumer stakeholders
Edwards et al.	2016	Intervention study with qualitative evaluation	Australia	93			Qualitative open-ended survey and thematic analysis	-Seven distinct phases of pregnancy journey for women with T1D: contemplation, pregnancy planning, conception, pregnancy, delivery/birth, and motherhood or pregnancy loss. - Eight themes in each stage: identity, relationships, physical, mental, knowledge, impact, medicalistion and balancing. Shifting priorities in each phase; information gathering varied between medical and non-medical (social media/peer support) based on perceptions of support from their HCPs. - Whilst medical intervention was expected and reassuring, peer support was more reassuring in the pre-contemplation/contemplation stage of pregnancy - Women are seeking advice from non-mainstream sources (peer support instead of PPC), with connections with other women with diabetes and pregnancy lived experience the most valuable process for women contemplating pregnancy with diabetes.		Very involved - thematic analysis of women with diabetes/their perceptions of support through pregnancy journey
Forde et al.	2016	Systematic review	UK	0 (however paper examined interventions with consumer involvement)			Meta Synthesis SLR	- Uptake on PPC is limited in women with T2D – reported lack of engagement with PPC as they were not informed about its importance - Women mainly informed by personal knowledge of reproduction needs, their relationship and communication with their HCPs, and system-level interaction. - Receptiveness to guidance for PPC was determined based on how HCPs communicated with the women, with many reporting they felt communication from HCPs was stigmatised by consumer age or obesity, and many felt that focus was on diet and exercise rather than the relationship between diabetes and pregnancy and its benefits. - Many consumers voiced strong emotions of guilt, distress and anxiety about their reproductive capacity and risks, as well as feeling a lack of control over their pregnancy due to medical intervention.		Not involved directly with study, however study examined literature where consumers provided previous experiences. Data from seven studies which interviewed 28 T2D women for their experiences
Grady et al.	2016	Intervention study with qualitative evaluation	USA	147			Self-report online survey with linear regression analysis	- Study examined the relationship between women’s locus of control, self-efficacy, and outcome expectations of preconception counselling. - Self-efficacy was positively associated with women’s expectation of preconception counselling, but self-blame for sub-optimal diabetes management was inversely related. - Fifty-seven% of consumers knew another women with diabetes who had been pregnant who they had met online. Social media is a valuable tool for sharing lived experiences, can increase confidence in women in their ability for a healthy pregnancy, and can supplement information provided by HCPS.		Minimal involvement - responses from online self-report survey with no follow-up or space for feedback.
Nwolise et al.	2016	Systematic Review	UK	0 (however paper examined interventions with consumer involvement)			Systematic Literature Review	- PPC interventions mainly delivered via HCPs, with eHealth and digital interventions significantly reducing adverse outcomes in diabetes and maternal health, and significantly improving knowledge and attitudes towards seeing PPC. - Consumers are proactively seeking information in the privacy of their own home, in their own time. Incorporation of health interventions into technology (such as social media and apps), is important as this is seen as highly empowering for consumers in making informed decisions about their care - Reverse digital divide also confirms CALD and low income consumers fastest growing users of mobile phones/apps - Health programs need to be developed so they can be delivered outside of traditional clinical setting, assisting in common barriers such as lack of time, lack of resources for HCPs to deliver, and distance/commute to appointments		Not involved directly with study, however study examined literature where consumers provided previous experiences.
Earle et al.	2017	Systematic review of qualitative studies and intervention study	UK	12			Mixed methods - a systematic review and qualitative interviews.	- Three main themes emerged for what consumers want from PPC: (1) effective communication/relationships, (2) provision of individualised care and (3) the co-ordination and organisation of care. -There is a lack of clarity over what PPC comprises and how it is perceived, as well as who is responsible for the delivery of this to women (which becomes more unclear for different types of diabetes). - Quality of care is dependent on enabling women as partnership in the form of care, and establishing trusting relationships on a partnership approach with HCPs, focusing on positive change rather than negative outcomes. -The results show that uptake of PPC is influenced by factors such as the complexity of pregnancy planning, expertise of HCPs and the quality of relationships with HCPS for consumers. Reframing PPC to place a greater emphasis on pregnancy planning, fertility and contraception would remove some of the existing barriers to uptake of care.		Involved - consumers were part of interviews for qualitative analysis. Study commented that their participation rate of 12 fell short from their prior sample size of 48
Nwolise et al.	2017	Intervention study protocol with qualitative evaluation	UK	0 (however paper is a protocol for setting up consumer intervention, with recommended sample size of 12)			Mixed methods study design - app usage and semi-structured interviews using Normalisation Process Theory (NPT)	- Paper aimed to explore the feasibility and acceptability of the Preconception and Diabetes Information (PADI) app to improve preconception care knowledge and attitudes in women with diabetes. This is the first study that examined PPC via an app and its engagement potential. - Consumer working group wanted an easily accessible and comprehensive PPC info that uses positive and supportive language. They wanted information should extend beyond PPC, and should include info on pregnancy and postpartum with diabetes. Suggestions included a blood glucose diary feature, reminders, graphs and a user-friendly design.		Very involved - consumer working group as part of intervention development and in proposed intervention design.
Wotherspoon et al.	2017	Intervention study with qualitative evaluation	UK	11			Qualitative study, semi-structured interviews, data analysis/thematic analysis methods.	- Study explored women’s knowledge on pre-eclampsia and a proposed screening test intervention for women with diabetes to raise awareness in PPC setting. - Women were happy to receive more precautionary information about conditions during pregnancy, as they had previously sought information from their own searches. They would prefer to know for preventative measures, even if the content is stressful. - Consumers felt their appointments for PPC were rushed, and did not allow time to ask questions about risks and decisions of their care. - Consumers felt supported by their diabetes team, but when being relayed to other clinical teams for PPC support they received less information on their care.		Involved - consumers were part of focus groups and interviews which were then analysed to determine study findings.
Sina et al.	2018	Narrative review of qualitative studies and intervention study	Australia	15 interviewed			Narrative literature review, chart review of outcomes, interview surveys, focus group, qualitative analysis and intervention strategy development	- A management strategy for pre-pregnancy was developed in an aim to improve outcomes and a reduction in poor diabetes management. This included multifaceted educational programs for both women and HCPs, which in turn resulted in increased contraception update. - Whilst 50% of pregnancy among women with pre-existing diabetes are unplanned, many had non- engagement with PPC due to lack of information/knowledge of the benefit of PPC, information overload and work commitment. Women also stated contraception barriers in lack of knowledge on emergency options, and myths and misconceptions from partners. - Lack of knowledge in relation to diabetes management/limited access to PPC was a main challenge for both women and their associated HCPs. Recommendations to HCPS to improve engagement include a better referral process between primary and secondary care and improved advertisement and awareness of services. - Social media engagement has been a demonstrated tool for raising and updating women’s knowledge and contraception update (particularly for low socio-economic/education audiences).		Involved - focus groups and interview in phase iii and iv of intervention to develop strategy using Delpha approach.
Britton et al.	2019	Intervention study with qualitative evaluation	USA	55			Cross-sectional, descriptive survey	- Study tested the association of perceptions and contraceptive use in postpartum period for women with pre-gestational DM. -Over half of consumers used procedural/prescription contraception - those who perceived contraception use and preconception care to be beneficial were more likely to use procedural/prescription contraception. -Consumers with diabetes-related complications were no more likely to think about diabetes when making contraception decisions (compared to those with no complications). - Over a third thought it was harder to get pregnant with diabetes, and most cited reasons for not using contraception such as fear of side effects, their partner/family does not support contraception, or they were planning to start planning for pregnancy later -Women’s perceptions on benefits of contraception and PPC may have implications on if use aligns with reproductive goals/optimises future pregnancy outcomes. They may delay PPC if they fear HCPs will disapprove of their pregnancy.		Involved- consumers completed a survey, but there was minimal follow-up or chance to participate in study further.
Forde et al.	2019	Intervention study with qualitative evaluation	UK	30			Qualitative in-depth interviews and thematic framework analysis	-Seven themes expressing factors that mediate reproductive behaviour and care in women with T2D were identified at the patient, professional and system levels. T2D generally perceived negatively by the women and HCPs. Many consumers reported feeling stigmatised by HCPs/family/friends for getting T2D (and that it was a lesser form of diabetes compared to T1D), resulting in negative self-image, shame, self-blame and guilt. Many did not disclose their condition as a result, and these negative perceptions influence health-seeking behaviours with tendency to disengage when communicating with HCPs. Many consumers indicated they would sort their diabetes health out once they had become pregnant, however those that did access PPC found it helpful, informative and personalised. - Pregnancy was rarely featured in their follow-up care, and PPC info provided was deemed uninformative and did not connect with consumer’s pregnancy planning or contraceptive use. There was a lack of awareness on PPC needs of population/communication between groups was unhelpful in finding reproductive intentions. - The themes also reveal a lack of systemic processes to incorporate pre-pregnancy care into the care of women with T2D, and consequently, HCPs in primary care have limited capacity to provide support.		Quite Involved - in-depth interviews with consumers to deliver findings via thematic analysis
Leow et al.	2020	Intervention study with qualitative evaluation	Malaysia	67			Observational, cross-sectional survey via self-administered questionnaire	-Consumers’ perception of PPC was poor - they had an inadequate knowledge of the effectiveness of their current contraceptive practice in relation to their intentions for further pregnancy and their self-perceived risk in case of future conception. - Most consumers were aware of family planning (as they had already completed their family and were after contraception), but not PPC - they were no longer interested in PPC. However, there was limited understanding on the types of contraception and recommendations for consumers with diabetes.		Involved- consumers completed a survey, but minimal follow-up. Study indicated there were limitation as sample size/responses
Nwolise et al.	2020	Intervention study with qualitative evaluation	UK	10 Consumers (19 including clinicians)			Exploratory study (focus groups and interviews) with thematic data analysis. Co-design approach, where consumer take on role of experts based on lived experience.	- Study aimed to explore views and experiences of consumers/HCPs using mobile app for PPC. Both parties highlighted challenges/lack of consistency in current PPC services. Women also felt HCPs had unsupportive attitudes towards pregnancy and were only told of adverse outcomes. Consumers were anxious about pregnancy, and preferred to wait to engage with diabetes team until they knew the baby was healthy. However, majority of women who engaged with PPC said experience was sub-optimal as they felt they received minimal information about pregnancy planning/care, with focus primarily being on diabetes requirements. - The acceptability of tech and apps from both parties creates huge potential to change barriers to quality of care and service in PPC and improve maternal and foetal outcomes. Many consumers were already using apps to manage parts of DM, but reported lack of PPC apps. - Consumers found apps improved PPC knowledge as information was easily accessible, with clear messages and positive language. Consumers agreed convenience of PPC app would appeal to many and had potential to increase uptake/engagement. Women with DM have close relationship with phones, so an app with the required PPC content would be ideal to ensure consumers are receiving consistent information - removes barriers such as access only via clinic/appointment.		Very involved - this study focused on thematic analysis of women with diabetes and their feedback on PPC via app
Ayman et al.	2021	Intervention study with qualitative evaluation	UK	287 initially, 473 interim, 9 workshop			Survey, literature search and workshop	- A Priority Setting Partnership was established to set future research goals in diabetes and pregnancy, based on women with lived experience in diabetes and pregnancy planning/journey. - The highest ranked priorities according to women and HCPs were diabetes technology, best tests for diabetes during pregnancy, diet and lifestyle interventions for diabetes management during pregnancy, emotional and wellbeing needs, safe full-term birth, post-natal care and support, diagnosis and management late in pregnancy,, women’s labour and birth experiences and improving planning pregnancy. - Important issues were continuity of care and support, consistency in care standards and advice, joint decision-making (particularly in labour and birth) and burden for women of being diagnosed with/managing diabetes. -Consequences on women’s well-being and mental health at core of discussion at final workshop/impact of a ‘medicalised’ pregnancy, withdrawal of intensive clinical involvement postpartum and support needs of women if adverse outcomes in child (linked to diabetes).		Very involved- PSP was consumer-driven, with a steering group/representation for entre study. The findings based on the experiences and feedback of consumers used to deliver outcomes of study.
Forde et al.	2021	Intervention study with qualitative evaluation	UK	30			Qualitative in-depth interviews and thematic framework analysis	-Six themes demonstrated a multi-modal approach for improving uptake to PPC. These were coherence (enhancing understanding of reproductive needs for women and HCPs), cognitive participation (constructing positive messages and narrative for consumer), collective action (increasing visibility of PPC needs and integrating healthcare systems/support tech), and reflexive monitoring (multi-modal approaches to support systemised care). These 6 themes can be used to develop strategies to improve PPC and the specific needs of women with T2D. -Lack of understanding about the relationship between diabetes and pregnancy-women with T2D identified need for information about PPC in initial diagnosis education - Consumers felt their questions were not addressed, and reproductive needs were low priority for HCPs – there was a focus on glucose levels and weight loss/common stigma constructs of T2D reduced confidence in PPC. - Vital role of technology in providing accurate information on PPC, but needs to be framed in positive way with motivational narrative. Better marketing/promotion of services via leaflets, posters, social media etc., especially in structured education programs for T2D. Consumers also recommended a feedback mechanism for both consumers and HCPs to provide reinforcing messages to both.		Involved - consumers were part of interviews for qualitative analysis.
Nwolise et al.	2021	Intervention study with qualitative evaluation	UK	17			Mixed methods study design- app usage, semi-structured interviews and questionnaires, thematic analysis, descriptive statistics/ non-parametric tests analysis.	- A follow-up study to the above trialling the PADI app with consumers and their feedback - the first study of its kind for a PPC app. Results show the app has potential to improve PPC knowledge, attitudes and behaviours. - App was viewed as ideal means of PPC information because of accessibility/portability, and was viewed as comprehensive and informative with self-management features that empowered its users. They reported the knowledge gained reduced their previous anxieties about planning pregnancy with diabetes, and app usage assisted in facilitating discussion of PPC with clinical teams prior to conception. - There were some limitation with the BGL diary - most used app for PPC, and only periodically for BGL diary as it required manual entry and they already were recording this elsewhere. Diary also lacked options to email/download/send to clinic, or to add info such as insulin dosages. Life commitments such as work interfered with frequent use of app.		Very involved - the app/intervention was co-created by consumers as part of a working group, and this study assesses consumer intervention participation and feedback/follow up.

Next, a meta synthesis analysis of tabulated data summarized in the matrix (Table 2) was undertaken with a phenomenological approach [13, 14] to develop an intervention framework (Table 3). This framework was generated to demonstrate recommendations for consumer involvement based on identified key themes from each paper. Where a theme was clearly identified within the matrix (Table 4) (being presented across multiple papers, or as a key theme or outcome from a paper), it was added to the framework.

**Table 3:** Pre-pregnancy Care (PPC) consumer and community involvement (CCI)-Driven intervention Framework for women with Pregestational Diabetes

Framework Component	Justification
Consumers/Community as active stakeholders in research and clinical interventions, with representation in all components as required (minimum working operations and advisory group to meet throughout project, and active role in intervention/service development).	Improved PPC knowledge/attitudes/behaviours in previous studies where consumers were actively involved and had ongoing representation, as well as more positive engagement with HCPs and stakeholders involved due to being equals in knowledge partnership (6).
Communication – ensuring language used in development and implementation is empowering and positive for community, delivering a message of support and being treated as equal partners to HCPS due to their lived experience.	Studies highlighted increased engagement with HCPs and extended outcomes in relation to access of care when positive, empowering communication methods were used (2, 15, 24, 26).
Greater information on contraception options at an earlier age – connection with transitional/young adult clinic to provide open-access education and support options, rather than purely PPC.	Several studies highlighted the lack of information readily available to women in clinical settings regarding contraception options (34), or that young adults were sexually active without contraception and had not received any contraception information (33).
Technology – intervention development highly recommended in a digital space, with apps strongly endorsed by consumers.	This alleviates common barriers to PPC such as appointment overwhelm, consumers being unable to attend around work/life commitments, as well as ensuring continuity of information/care received through the resource (40). The same technology approach should also be implemented for consumers in the intervention working group to ensure accessibility.
Social media/peer support components – lived experience is a valuable currency for community and HCPs alike to strengthen interventions and engagement with healthcare services.	Inclusion of consumers from intervention working advisory group in social media/peer support components of intervention strengthens participation rates and engagement with consumer participants (18), as well as provides a holistic model of care to compliment what may include clinical/medical components of study (21)(Sparud-Lundin et al.).
Renumeration – consumers want to be acknowledged for the value of their lived experience and expertise for a project. As such, intervention project/service budgets should account for appropriate payment for community representatives in the working group, as well as payment of consumers who participate in the intervention itself.	Working group in study by Nwolise et al. (28) and Ayman et al. (22)(Priority Setting Partnership) where consumers had active and ongoing representation resulted in extensive findings and improved knowledge on successful future intervention development.

**Table 4:** Most common themes

Themes	Summary
Barriers to PPC	Too many appointments, difficulty balancing these with work/family, and a lack of knowledge on the services and alternatives available.
Negativity/stigma in care	Perceptions/attitudes/behaviours of HCPS.
Fear of losing a “normal” pregnancy journey	Extensive clinical intervention in care – women desire for their pregnancy to be celebrated, rather than medically critiqued.
Awareness of risk	Women are aware of risk, but prefer open communication with HCPs (partners in care to manage expectations.
Lived Experience	Women proactively seek out lived experiences for assurance and positivity – frequently through social media/peer support.
Accessibility	Women state that information on PPC (including contraception) needs to be more readily available and at earlier stages by HCPs (adolescent/ transitional clinics). However, cultural taboo regarding contraception needs to be carefully navigated for CALD audiences.
Digitization	Women are seeking alternative PPC through digital methods (i.e., apps, telehealth, structured peer support), allowing for greater accessibility and accuracy/quality of information received from HCPs.

## Results

A total of 660 hits were identified, with 29 of these fulfilling inclusion criteria (see Appendix 3 for study selection PRISMA flowchart). The main reasons for exclusion at the final screening stage were: papers focused on pregnancy or postnatal studies (*n* = 250 papers did not have consumer involvement or were not diabetes-related (*n* = 143), papers involved children/teens unrelated to contraception or pregnancy planning (*n* = 15) or papers involved women with GDM only (*n* = 250). Of the 29 included articles, six were systematic reviews examining PPC interventions [1, 6, 8, 15–17], whilst the remaining 23 were analyses of PPC interventions and consumer/HCP feedback on current services related to PPC [2, 9, 18–38]. Five of the 29 papers had a leading consumer focus [1, 2, 6, 9, 22], whilst Ayman et al. had a complete consumer focus as its centre thematic framework [22]. Studies ranged in publication from 2009 to 2021, with the majority of studies (*n* = 17) conducting their research in the United Kingdom [1, 6, 9, 15, 16, 19, 22, 24, 26, 28–30, 34–40], USA (*n* = 5) [17, 23, 25, 31, 33], greater Europe (*n* = 3) [2, 21, 32] and Australia (*n* = 3) [8, 18, 20]. Qualitative research methods via interviews, survey, or focus group were the methodologies used for papers that examined existing completed or proposed interventions [2, 6, 8, 9, 18–24, 26, 27, 32, 35–40], with several of these studies also incorporating quantitative methods and following a mixed-methods approach [15, 28, 30, 31, 34] to examine additional data for reporting specific psychological and clinical outcome measures.

The most common themes presented in the included 29 papers were (in summary):

These themes will be further elaborated in detail below:

*Barriers to PPC*: A key concern highlighted by consumers when reporting barriers to accessing PPC is the logistical barriers to appointments. In particular, the requirement for an increased frequency of appointments, along with face-to-face appointment requirements and travel which interrupted work and life commitments [6, 9, 26]. Many consumers recommended flexibility in appointments, particularly in relation to digital health platform offerings such as telehealth or app connectivity with health care professionals [28].

*Negativity and stigma in care*: Consumers actively highlighted that attitudes, language and assumptions of HCPs that they access in their journey to PPC (primary and secondary care) are a significant barrier to engagement and participation in PPC interventions, with over half of the studies examined reporting this as a key barrier to PPC access and engagement [8, 9, 24, 35]. In particular, studies that examined lived experience demonstrated the negative impact that poor communication (including negative attitudes, judgement, stigma, disempowering language and misinformation) and service from healthcare professionals can have on consumers’ self-efficacy and pregnancy planning [9]. Research also demonstrates that women in the pre-contemplation stages of pregnancy planning with pre-existing diabetes place a higher value on recommendations from other women with diabetes in comparison to recommendations from healthcare professionals [18].

*Fear of losing a “normal” pregnancy journey*: Consumers reported a disconnect between primary and secondary care experiences – that whilst they had frequent appointments across multiple services, there was a lack of continuity of care [35]. Many consumers stated that there was a focus from service providers on following procedures for high-risk pregnancy whilst being under time and resource constraints, whereas the women would have preferred more time to be taken to ask questions and celebrate the experience of pregnancy [36]. As a result, there was a clear presentation of previous interventions demonstrating women felt their pregnancies were very medicalised, with the lack of opportunity for their journey to be celebrated and recognised with the milestones of comparatively normalized pregnancy experiences.

*Awareness of Risk*: Consumers felt that HCPs need to establish trusting relationships with them even before attempting to engage with an intervention, as well as revise their perception of consumers – to be treated as equals in partnership of their healthcare needs, and having their lived experience acknowledged and positively framed [40]. Consumers noted several times across interventions that if a trusting relationship is already established with HCPs, they are more receptive to discussions regarding risks and adverse outcomes in unplanned pregnancy [2, 15].

*Lived Experience and Fear*: Consumers highlighted that the lived experiences of other consumers in having a successful pregnancy journey was vital to reducing fear and distress [18]. In fact, when many felt they had received a knowledge gap between clinical services (that it was not supportive enough), they found that peer support and social media helped to bridge the gap whilst strengthening their self-efficacy in diabetes and pregnancy management [21]. It was clear from across the studies that regardless of the type of intervention, consumers place great value on the lived experiences of others to inform their decision-making process and perceptions of their own pregnancy journey.

*Accessibility*: Consumers also reported health care professionals should be more aware of cultural and social barriers in accessing contraception to avoid unplanned pregnancy. This was particularly highlighted in the context of adolescent women with diabetes who may need access to contraception, but are either too embarrassed to ask healthcare professionals due to family, religious pressure or cultural taboo [19, 23]. Consumers also stated adolescents are under-reporting sexual activity to HCPs due to social desirability bias or they are attending appointments with parents who may not be aware of their child’s need for contraception. Therefore, consumers highlighted the need for maintaining confidentiality with regards to their sexual activity [33]. Women also highlighted that they were provided with contraception information in a clinical setting only at times they deemed stereotypical (of when it was expected i.e., age and/or marital status) and felt stigmatised by their partner or marital status [35]. However, consumers indicated that they required this information in relation to how it impacts diabetes management much earlier than clinically anticipated by their diabetes team, and in more detail than what is provided through primary care and GP services. A study conducted in the United Kingdom noted that women with Type 2 diabetes reported receiving no information on contraception options, including after an unplanned pregnancy with complications [34]. Many women reported a familiarity with base contraception options such as the oral contraceptive pill, however studies demonstrated a clear knowledge gap in relation to longer-acting contraception options (LARC) and hormonal contraception options, as well as clinically-reported health benefits and risks of each associated option [34]. Consumers particularly reported that they referred to sources such as the internet to review contraception options and risk, with many citing they had been influenced on their contraception options primarily by family, friends or peers [34, 36].

*Digitalisation*: Within more recent studies, particularly during the COVID-19 pandemic, consumers have highlighted that they are actively seeking PPC information from non-mainstream sources: outside of clinical settings [5, 41, 42]. In particular, technological support platforms such as social media and peer support delivered via multiple methods (face-to-face, online, phone etc.) are highlighted as fulfilling a knowledge gap that many consumers are experiencing due to disconnect between primary and secondary care [18, 21, 25], or the inconsistency of information provided in clinical settings between diabetes and antenatal teams [15, 26]. The value of peer support has briefly been explored in the context of a PPC intervention through an integrated care attempt in Sydney, Australia [8] and the Preconception and Diabetes Information (PADI) app trialled in the United Kingdom [29, 30].

These themes (summarized in Table 4) were then used to construct the following intervention framework:

## Discussion

This is the first study to examine the extent of CCI in an intervention and its development in relation to diabetes and PPC. Whilst studies demonstrate CCI in components and associated findings from their engagement in the study development, an analysis of the perspectives and themes presented by consumers in relation to successful intervention development and engagement with fellow consumers and community has not been conducted previously.

The main findings show that most studies held a structure which involved CCI providing feedback on an intervention following participation in it via surveys/focus groups, however there was minimal involvement from the women in development of the intervention and services. Therefore, qualitative data collection served purely to evaluate interventions after its delivery with minimal follow-up or scope for further developmental changes from community feedback. Mixed method approaches of surveying consumers, followed by focus groups or one-on-one interviews held the capacity to provide thematic, qualitative analysis, with hermeneutic-based studies [2, 8, 9, 18, 21, 26] delivering the most thorough analysis of consumer feedback through these methods. However, the studies by Lawrence et al., Forde et al., Holmes et al. and Earle et al. are also commended for their proactivity in application of consumer feedback. Therefore, there is untapped potential for development of interventions with consumers at the forefront from project inception and ongoing representation through the project lifecycle – development of a “best practice” framework to ensure maximum engagement potential with target consumers is highly recommended. The recorded levels of involvement in the study matrix indicates that few pre-pregnancy interventions have been created with consumers and community as active and ongoing stakeholders – from development to delivery. Exceptions can be found including the projects by Nwolise et al., where across two papers consumers were an active component from project conception to delivery, and for the study by Ayman et al. where a complete consumer steering group (Priority Setting Partnership) was established and maintained for the entire study duration and beyond. Both of these studies held a unique value proposition in the context of social marketing and engagement tools for CCI – the project by Nwolise et al. in the development of an app for preconception care, and the study by Ayman et al. in the establishment of a formalised steering group with opportunity for ongoing formalised professional development for consumers. This links to the notion that consumers are driven to assist with interventions when they can clearly see the benefit for themselves, as well as the targeted community cohort.

Existing studies in the context of PPC interventions demonstrated a clinical focus, rather than highlighting the consumer and community perspective as a main pillar of knowledge for intervention development. Consumers and community have been under-involved in the majority of previous developments and implementation of interventions for women with diabetes and pregnancy. Minimal CCI and limited collaboration between consumers and HCPs directly links to the reported barriers to accessing PPC; negativity and stigma in care from the behaviours, attitudes and perceptions of HCPs; frequency of appointments being a barrier to work and family commitments; fear of losing a “normal” pregnancy journey; awareness of risk but willingness to discuss if consumers have trust in HCPs; inaccessibility to PPC and contraception in context of CALD and digitisation of alternative PPC information resources including peer support and social media. To counteract the barriers to successful intervention development, ongoing consumer representation as stakeholders in interventions is essential to the longevity of intervention outcomes. Examining consumer perspectives of these interventions is a potential untapped research area due to the minimal research literature and data, as highlighted during this study. Including these perspectives in the development and implementation of interventions is vital to improve intervention outcomes.

A key recommendation of this study, as highlighted in the CCI framework, is offering flexibility in appointments/clinical reporting through mixed-methods of delivery (digitised methods such as an app, telehealth or other means as recommended by consumers). The hybrid delivery options will depend on the needs of the person with diabetes and clinical service delivery requirements. This recommendation is not offered to replace face-to-face offerings, but rather establish transparent and equitable access to resources for diverse consumer audiences. To establish varying types of accessibility to clinical teams and appointments, capturing of HCP perspectives on services, as well as creation of professional development resources to assist in transition to digital platform offerings for HCPS, were highly recommended by consumers.

It is also pivotal for future interventions to have relationship development and establishment of trust in a clinical setting as a main pillar of the study in order to maintain its success and engagement with target consumers. In order to accomplish this, it is recommended for additional professional development to occur which is tailored to diabetes and PPC for HCPs, with an extension tailored to general practitioners and other primary carers – so as to provide an integrated system of continuity of care for the community.

Adding a layer of peer support via a technological intervention component for PPC has not yet been examined. Consumers and community are actively accessing technology for their healthcare needs, and many interventions having a primary clinical/hospital location are potentially missing an untapped market to engage with consumers who most need PPC access. It is important to note that since this review was conducted, more research has been published by Murphy et al. on access to technology during pregnancy including continuous glucose monitoring (CGM) [43] Future studies consider the integration of technology such as access to wearable devices when developing interventions.

This study also demonstrates a clear need for provision of contraception information framed in a setting outside of preconception care that can be confidentially available but easily accessible by younger people with diabetes. This accessibility of information is important to engage with target consumer cohorts from a CALD background. In the same context of provision of contraception information in a timely manner, there is also evidence of women with diabetes not being provided with adequate and accurate contraception options. This hinders the opportunity to enable them to make an empowered contraception decision that most benefits them and their family planning health journey.

The studies examined also highlight how important it is to accurately provide information (supported by mixed-media delivery) on contraception options available in a formal setting, with clear demonstration of benefits and risk of each option in relation to diabetes and pregnancy planning management. This is especially important in the context reported by consumers of potential miscommunication between primary and secondary care [2, 8] and contraception education (such as not all options being explored, lack of understanding of hormonal contraception/risk of some contraceptive options and impacts on diabetes management).

Limitations of this study included the limited number of pre-pregnancy intervention studies (in comparison to many diabetes and pregnancy intervention studies), a reduction in more recent papers to analyse during development of the matrix due to publishing reductions from the global pandemic crisis, as well as differing language used for scoping search terms. Scoping search terms were limited to predominately European or American terminologies in order to capture eligible intervention studies. Australian-only search terms were tested, with very limited results in comparison to using more broader global maternity and health terms when conducting searches. When more global language was used in searches, Australian studies were still captured and included in the search results due to other key consistent language mesh search terms used. However, it should be noted that due to the inclusion criteria requirement of studies being reported in English, there may be selection bias of included articles. Papers were assessed using a quality assessment tool for risk of bias, with 8 studies (Appendix 2) displaying a high risk for selection bias in the participants selected (primarily due to the women being those engaged with the services currently offered).

How to best identify the target audience/consumer in terms of inclusive language – whether terms for non-cisgender identifying women was raised, or whether terminology should be updated to reflect people with female reproductive organs. As literature databases predominately used medical identifiers for their mesh terms, woman/female was used to capture the most accurate intervention studies. However, it is important for future studies to consider the terminology used around gender and reproductive status to ensure it is inclusive and representative of diverse target cohorts. It is also important to note that at the time of literature searches and data analysis, the preferred terms for people with diabetes involved in studies was “consumer”, however recent studies are now recommending future researchers to use the term “community” in order to be more inclusive and reflect on the holistic nature of the people involved being more than a consumer, but an all-rounded component of the studies and beyond. This also applies in context of “consumer engagement” – “community involvement” is now preferred people-centred-care language [44, 45].

## Conclusion

Applying the *Pre-Pregnancy Care (PPC) CCI-Driven Intervention Framework for Women with Pregestational Diabetes (*Table 2*)*, researchers and clinicians will be able to develop an engaging, empowering intervention with consumer endorsement, increasing successful outcomes based on components of prior studies with consumer involvement.

In order to be most engaging with people with diabetes planning pregnancy it is recommended for future interventions to consider allocating funding and resources in their projects to include consumers in all stages of intervention development. It is imperative that additional resourcing for translation/delivery through community collaboration is established for future projects to ensure that CALD communities have more equitable representation in intervention studies. It is also recommended based on previously successful consumer-driven studies for future interventions to develop and implement an integrated consumer-driven PPC app with long-term usability and accessibility options moving forward in healthcare systems impacted by global pandemic resource and information shortages. This is to ensure equitable access of information and support for women with diabetes, ongoing professional development and resources for HCPs to learn and adapt to new technologies to support consumers, and additional communication coaching for HCPs to ensure language and attitude presented to consumers is aligned with a healthcare model of co-creation and collaboration.

## Data Availability

The datasets analysed during the current study are available from the corresponding author on reasonable request, and the literature matrix is included.

## Abbreviations

CCI: Consumer and Community Involvement
DCAPP: Diabetes Contraception and Pre-Pregnancy Program
HCP/s: Health care professional/s
PPC: Pre-Pregnancy Care
PPM: Pre-Pregnancy Management
T1D: Type 1 diabetes
T2D: Type 2 diabetes
